# The Differences in the Levels of Oxidative Status Marker and Soluble CD95 in Patients with Moderate to Severe COPD during an Exacerbation and a Stable Period

**DOI:** 10.1155/2021/2105406

**Published:** 2021-12-09

**Authors:** Svetlana Soodaeva, Nailya Kubysheva, Igor Klimanov, Alexey Shutov, Tatyana Eliseeva, Viktor Novikov, Klavdiya Kontorshchikova, Dmitry Novikov, Ildar Batyrshin

**Affiliations:** ^1^Pulmonology Scientific Research Institute under FMBA of Russia, Orekhovyy Bul'var 28, Moscow 115682, Russia; ^2^Kazan Federal University, Kremlyovskaya St, 18, Kazan 420000, Russia; ^3^Federal State Budgetary Educational Institution of Higher Education Privolzhsky Research Medical University, Minin and Pozharsky Square 10/1, Nizhny Novgorod 603005, Russia; ^4^N.I.Lobachevsky Nizhny Novgorod National Research State University, Gagarina Avenue 23, Nizhny Novgorod 603950, Russia; ^5^I.N. Blokhina Research Institute of Epidemiology and Microbiology, Malaya Yamskaya St., 71, Nizhny Novgorod 603950, Russia; ^6^Instituto Politécnico Nacional, Centro de Investigación en Computación (CIC-IPN), Av. Juan de Dios Bátiz, Esq. Miguel Othón de Mendizábal S/N, Gustavo A. Madero, 07738 Mexico City, Mexico

## Abstract

Studying the features of changes in markers of oxidative stress (OS) and inflammation indicators in COPD patients depending on the degree of bronchial obstruction is one of the priority directions for improving the prognosis and monitoring of the course of this pathology. We conducted a comparative investigation of changes in markers of OS and apoptosis at the systemic and local levels in patients with moderate to severe COPD during exacerbation and stable phase. 105 patients with COPD aged 46-67 and 21 healthy nonsmoking volunteers comparable in age were examined. COPD patients were divided into four groups: moderate COPD (GOLDII) during the exacerbation (GOLDIIex, *n* = 25) and in the stable phase (GOLDIIst, *n* = 27), severe COPD (GOLDIII) during the exacerbation (GOLDIIIex, *n* = 29), and in the stable phase (GOLDIIIst, *n* = 24). We studied the levels of such lipid peroxidation (LPO) products as diene conjugates (DC) and Schiff bases (SB) and parameters of induced chemiluminescence (Imax, total light sum-S, Imax/S) in blood serum, as well as sCD95 concentration in blood and exhaled breath condensate (EBC). The relationship between the values of the OS system indicators with sCD95, as well as with the parameters of lung function, was investigated. Multidirectional changes in OS indicator levels in COPD patients depending on the severity of obstructive airway disorders have been established. The maximum values of DC (0.26 ± 0.046 RU), Imax (0.265 ± 0.19 RLU), and Imax/S (0.13 ± 0.05) were typical for patients with moderate COPD, while the highest SB level (5.7 ± 2.3 RU) was observed in severe COPD during an exacerbation. The exacerbation of the disease was characterized by an increase in DC concentration in both GOLDIIex (0.26 ± 0.046 RU) and GOLDIIIex (0.209 ± 0.02 RU) compared to the stable moderate and severe COPD (0.202 ± 0.028 RU and 0.19 ± 0.03 RU, respectively, *p* < 0.05). The established decrease in high values of DC, Imax, Imax/S, and sCD95 and an increase in SB concentration in GOLD III can serve as quantitative indicators of the prognosis of the severity of the disease. The serum concentration of sCD95 in GOLDIIex (366.4 ± 70.5 U/ml) and GOLDIIst (361.4 ± 72.8 U/ml) did not differ from the control group (393.7 ± 80.9 U/ml, *p* > 0.05). In patients with FEV1 < 49% during the exacerbation and stable phase, the serum levels of Imax/S (0.058 ± 0.01 and 0.062 ± 0.01) and sCD95 (318.2 ± 66.3 U/ml and 321.4 ± 42.5 U/ml) were lower than the values of healthy volunteers (0.08 ± 0.01 and 393.7 ± 80.9 U/ml, respectively, *p* < 0.05). A positive correlation between sCD95 concentration and airway obstruction degree in all examined COPD patients was established. The revealed numerous associations between sCD95 and OS marker levels in GOLDIII indicate a relationship between systemic radical stress and apoptosis processes both in the respiratory tract and the whole body under conditions of severe inflammation. The established correlations between the values of DC, Imax, and sCD95 in the blood serum and the lung function parameters in all studied patients allow us to consider these indicators as additional prognostic indicators of disease intensification. Our work results help clarify the participation and detail of FRO and apoptosis processes in developing pathophysiological features in moderate to severe COPD in different periods and, accordingly, improve the efficiency of diagnosis and treatment of the disease.

## 1. Introduction

Local and systemic inflammation in COPD is closely related to the intensification of free radical oxidation (FRO) processes and the development of oxidative and nitrosative stress [1–5]. The leading role of oxidative (OS) and nitrosative stress (NS) in the damaging effect on almost all lung structures, especially in the formation of lung tissue remodeling, is well known [1, 5].

Several studies have shown an increase in OS and NS marker concentration in various biological environments in COPD patients [1, 2, 4]. An increase in H2O2 concentration and the total oxidative status (TOS) in the EBC was revealed in this disease [6–8]. In some works, an increase in Fe2+ level in the respiratory tract was noted [9]. The interaction of hydrogen peroxide and divalent iron in the Fenton reaction can lead to the overproduction of extremely reactive hydroxyl radicals, which initiate the processes of FRO and lipid peroxidation (LPO). An increase in lipid peroxidation products such as MDA, 4-Hydroxy2-nonenal, and 8-isoprostane has also been found in serum, EBC, and sputum in COPD [8, 10–13].

Along with an increase in FRO indices, it was found that in patients with this disease, the activity of antioxidant enzymes, such as SOD, catalase, and glutathione peroxidase, as well as nonenzymatic antioxidant concentration (vitamins A and C, glutathione, etc.) decreases [14, 15]. However, several other studies have obtained opposite data on OS indicators in COPD. For example, an increase in enzymatic antioxidant activity, the absence of an increase in the level of lipid peroxidation products in patients with this disease has been shown [16, 17].

A significant proportion of OS and NS studies in COPD are devoted to investigating biomarkers reflecting the intensity of FRO directly in the respiratory tract: exhaled breath condensate (EBC), sputum, and BAL fluid [10–12, 18–20].

Currently, information is accumulating on circulating systemic OS markers associated with various pathophysiological disorders in COPD patients [21]. However, the feature changes in the systemic indicators of radical stress in COPD depending on the severity and period of the disease have not been sufficiently studied. The activity of OS processes is often researched by the lipid peroxidation indicators, such as MDA and isoprostanes. At the same time, it is important to study the intensification of lipoperoxidation reactions by the values of the initial and end products of LPO—diene conjugates (DC) and Schiff bases (SB). It is also relevant to determine the DC to SB (DC/SB) ratio, which allows you to define the direction and expressive of lipoperoxidation processes stages. There are few studies of these molecular products in patients with COPD, depending on the severity and period of the disease. There are few studies of these molecular product concentrations in COPD patients depending on the severity and period of the disease.

It should be noted that one of the most significant ways to investigate FRO reactions is to determine the potential ability of lipid substrates to form free radicals, which can be detected using chemiluminescence (CL) analysis. This method allows us to comprehensively evaluate both the prooxidant and antioxidant properties of the biosubstrate [22–24]. Given that there has been a growing interest in a complex assessment of the oxidant/antioxidant system in COPD, using this integral method to determine OS markers in developing this disease is essential.

In addition to the study of FRO, to identify the mechanisms of pathophysiological processes in COPD, special attention is paid to investigating the relationship between OS indicators and inflammation markers, such as cytokines and soluble differentiation molecules (sCD). Earlier in our works, we showed the role of several soluble forms of membrane molecules in the mechanisms of development of systemic and local inflammation in patients with this disease [3, 4, 25, 26]. In particular, changes in soluble CD95 (sCD95) concentration in serum and EBC were detected in moderate and severe COPD patients during an exacerbation [3]. The sCD95 molecules are one of the apoptosis-specific markers and play an essential role in developing and regulating inflammatory processes in the airways and at the systemic level [3, 27]. For a more detailed study of the pathogenetic mechanisms of COPD progression, it is necessary to comparatively research changes in FRO markers and sCD95 levels in the blood and airways in patients with COPD, depending on the stage and period of the disease.

The study is aimed at investigating changes in the concentrations of DC, SB, and CL parameters in the blood serum and the level of sCD95 in the circulation and exhaled breath condensate in moderate to severe COPD patients during the exacerbation and stable phase.

Analysis of the relationship between OS and sCD95 markers, as well as these indicators and lung function parameters, will help clarify the involvement of FRO and apoptosis processes in the development of pathophysiological features of COPD and improve the diagnosis efficiency and therapy of the disease.

## 2. Materials and Methods

The study included 126 people: patients with COPD (*n* = 105) and healthy nonsmoking volunteers (*n* = 21).

The diagnosis of COPD was defined and classified according to the criteria of the Global Initiative on Chronic Obstructive Pulmonary Disease (GOLD) [28]. The COPD diagnosis was established based on largely irreversible airway obstruction with an improvement in FEV1 < 12% after inhalation of 400 *μ*g salbutamol. Lung function was measured again 15-20 minutes after inhalation of the bronchodilator to assess bronchodilator-induced bronchospasm reversibility.

COPD patients (*n* = 105) were divided into four groups: patients with moderate COPD (GOLDII) during the exacerbation (GOLDIIex, *n* = 25), patients with moderate COPD in the stable phase (GOLDIIst, *n* = 27), patients with severe COPD (GOLDIII) during the exacerbation (GOLDIIIex, *n* = 29), and patients with severe COPD in the stable phase (GOLDIIIst, *n* = 24).

The presented study is a pilot, so we did not use traditional approaches to calculating the sample size [29]. Currently, there are several practical rules according to which the group size in the pilot study is from 15 to 35 people [30, 31].

The study was implemented based on the principles of the Helsinki Declaration. Written informed consent was obtained from all participants. The study was approved by the Ethics Committee of the Pulmonology Research Institute, Moscow, Russia (the protocol number N 05-19 от 16.10.2019).

The study included COPD patients meeting the following inclusion criteria: age over 40 years, active or ex-smokers (smoking index (IC) ≥ 10 pack-years), exacerbation of COPD and stable period, and evidence of obstructed lung function (postbronchodilator FEV1 < 80% and FEV1/FVC < 70%) according to the GOLD [28]. An exacerbation was defined as a change in the symptoms of a cough, expectoration, and dyspnea beyond the daily variation and required changes in therapy in COPD patients.

The exclusion criteria were the following: asthma and other allergic diseases, pneumonia, history of congestive heart failure, severe arterial hypertension, diabetes, and conditions requiring the long-term use of systemic corticosteroids.

The control group included healthy nonsmokers with similar gender and age indicators who did not take any medications. Healthy subjects underwent a medical examination at the clinic and were randomly selected as a control group. The participants in the healthy group had no diagnosed respiratory diseases, diabetes, coronary heart disease, malignancies, or connective tissue diseases.

A pulmonary function study was carried out on a computer Spirograph “SpiroLab III” (Italy) for the evaluation of the FEV1, FEV1/FVC, and the parameters of inspiratory capacity (IC).

### 2.1. Serum and Exhaled Breath Condensate Preparation

Blood samples were obtained in the morning on an empty stomach from the middle cubital vein, immediately centrifuged at 3000 rpm for 10 minutes, and then extracted. Serum samples were frozen at -40°C.

EBC was collected using the RTube and following the guidelines for EBC by the ERS/ATS Task Force [32]. All patients were asked to refrain from drinking any liquid (except water) for 2 hours before the collection of EBC. To avoid oral or nasal contamination, the patients were asked to rinse their mouths with freshwater before collection and to wear a nose clamp during collection. The donors were asked to use tidal breathing into the mouthpiece for 10 minutes. After the 10-minute period of breathing is over, the samples were immediately stored and cooled to -40°C.

### 2.2. Measurement of the Diene Conjugates and Schiff Base Concentrations

The concentrations of DC and SB were determined as described in [33, 34] spectrophotometrically on a PerkinElmer LS-50 spectrophotometer. The levels of these LPO products were expressed in relative units (RU).

### 2.3. Chemiluminescence Analysis

To determine the intensity of free radical processes in blood serum, we used the CL method induced by hydrogen peroxide with ferrous sulfate. The measurements were carried out on a Dynatech chemiluminometer (Germany).

The following CL indicators were analyzed:

Imax (relative light units (RLU)) is the maximum value of the CL outbreak intensity, reflecting the biological system's potential ability to develop FRO processes.

S (total light sum) is the area under the CL response curve, which characterizes the FRO activity and is inversely proportional to the antioxidant activity (AOA).

Imax/S is the ratio that characterizes antioxidant activity (AOA) of the reaction system.

### 2.4. Measurement of sCD95 Concentration

The levels of soluble CD95 molecules in the serum and the EBC were determined by enzyme-linked immunosorbent assay (ELISA) using an ELISA reader (Multiskan MS, Labsystems, Finland) wavelength of 405 nm. In determining the content of soluble CD95 molecules, we used goat polyclonal antibodies against PBMC superficial antigens and mouse monoclonal antibodies ICO-160 against the CD95 antigen conjugated with horseradish peroxidase. The results were expressed in conventional units (U/ml).

### 2.5. Statistical Analysis

The statistical analysis was carried out using the Statgraphics Centurion software package, v.9. The data were presented as the mean ± SD. To determine the distribution normality, the Shapiro-Wilk test was used. The student's *t*-test performed further analysis. To calculate the correlation coefficient (*r*), the Pearson correlation test was used. The statistical significance level was considered to be *p* < 0.05.

## 3. Results

**Figure 1:**
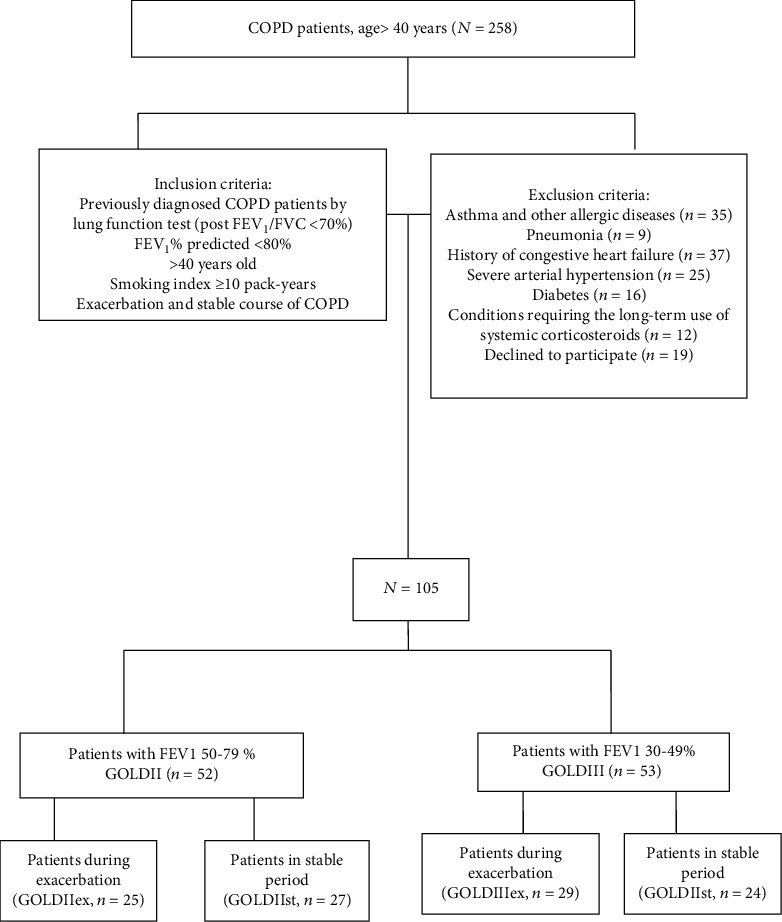
The flowchart of the study population. COPD: chronic obstructive pulmonary disease; FEV1: forced expiratory volume in 1 s; FVC: forced vital capacity.

Figure 1 shows a block diagram of patient recruitment. A total of 258 people have successfully passed spirometry. Out of 258 patients, 153 people did not participate in this study according to the exclusion criteria. Therefore, 105 patients were recommended for further examination.

The demographic and clinical characteristics of individuals are shown in Table 1. There was no significant difference between the groups by age. In all groups, the majority of subjects were men. Spirometric values such as FEV1%, FEV1/FVC ratio, and IC were significantly lower in COPD patients compared to the control group (*p* < 0.01). Lung function parameters in GOLDII exceeded similar values in patients with severe COPD (*p* < 0.01). The exacerbation of the disease was characterized by a decrease in the values of the studied spirometric parameters compared to the stable phase in both GOLDII and GOLDIII (*p* < 0.05).

### 3.1. The Serum Concentration of Diene Conjugates in COPD Patients

The DC level was increased in all COPD patients compared to the control (*p* < 0.01) (Table 2). The maximum value of these LPO products was observed in GOLDIIex relative to all the examined patients. The stable period of the disease was characterized by a decrease in DC level compared to during exacerbation in both moderate and severe COPD (*p* < 0.05).

### 3.2. Schiff Base Concentration in COPD Patients

The SB concentration in all the examined patients exceeded the values of the control (*p* < 0.05) (Table 2). The highest SB level was found in GOLDIIIex relative to controls and patients with moderate COPD (*p* < 0.05). The concentration of these LPO end products in patients during the exacerbation did not differ from analogous values in the stable phase in both moderate and severe COPD (*p* > 0.05).

### 3.3. Index of DC/SB in COPD Patients

To identify the expressive of the initial or final stages of LPO, the DC/SB ratio was determined. The value of the DC/SB index in patients with moderate COPD was significantly higher compared to the control (*p* < 0.05) (Table 2). At the same time, there was a decrease in DC/SB values in severe COPD both during exacerbation and stable periods relative to healthy volunteers and GOLDII patients (*p* < 0.05). There were no differences in DC/SB level depending on the periods of the disease in the examined individuals.

### 3.4. Chemiluminescent Parameters in the Blood Serum in COPD Patients

The Imax and S values were increased in all examined COPD patients in comparison with healthy volunteers (*p* < 0.05) (Table 2). At the same time, the high Imax level gradually decreased as the severity of the disease increased. The maximum CL intensity in the serum was recorded in GOLDIIex and was higher than in severe COPD patients during exacerbation (*p* = 0.01) and in the stable phase (*p* = 0.01).

The highest light sum (S) values were found in GOLDIIIex patients. This indicator's value was lower in all examined COPD patients in the stable phase than during exacerbation (*p* < 0.05).

The ratio Imax/S (AOA) was higher in moderate COPD compared to control (*p* > 0.05) (Table 2). The level of Imax/S (AOA) in severe COPD was lower than in healthy volunteers and all GOLDII patients (*p* < 0.05). There were no differences in Imax/S (AOA) value depending on the period of the disease.

### 3.5. sCD95 Concentration in Blood Serum and Exhaled Breath Condensate in COPD during Exacerbation and Stable Periods

**Figure 2:**
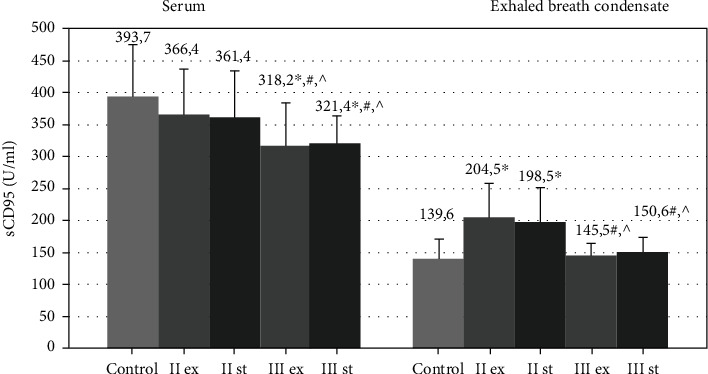
The concentration of sCD95 molecules in blood serum and exhaled breath condensate in COPD patients during the exacerbation and the stable period. Data are presented as mean ± SD; control: healthy nonsmoking volunteers; II: moderate COPD; III: severe COPD; ex: exacerbation; st: stable phase. ^∗^*p* < 0.05 versus healthy nonsmokers; #*p* < 0.05 versus patients with moderate COPD during the exacerbation; ^*p* < 0.05 versus patients with moderate COPD in the stable period.

The serum level of sCD95 in patients with moderate COPD did not differ from the level of healthy nonsmoking volunteers (393.7 ± 80.9 U/ml, *p* > 0.05) (Figure 2). However, the concentration of these molecules in severe COPD during the exacerbation (318.2 ± 55.4 U/ml) and stable period (321.4 ± 42.5 U/ml) was statistically lower than in healthy nonsmokers (*p* < 0.001), GOLDIIex (366.4 ± 70.5 U/ml), and GOLDIIst (361.4 ± 65.4 U/ml, *p* < 0.001).

The level of sCD95 in EBC was higher in patients with moderate COPD both during the exacerbation (204.5 ± 41.5 U/ml) and the stable period (198.5 ± 38.3 U/ml) than in healthy volunteers (139.6 ± 31.2 U/ml, *p* = 0.001). The endobronchial concentrations of sCD95 in GOLDIIIex (145.5 ± 19.5 U/ml) and GOLDIIIst (150.6 ± 23.3 U/ml) were significantly lower compared to the GOLDII patients (*p* < 0.001). They did not differ from the levels in the control group (*p* > 0.05).

### 3.6. The Correlations between Oxidative Stress Indicators, sCD95 Levels, and Lung Function Parameters

We found correlations between the values of spirometric indicators and the studied OS markers in COPD patients (Table 3). A negative association between DC level and FEV1 and FEV1/FVC was revealed in all COPD patients. The inverse correlation was established between SB concentration and FEV1/FVC (%), IC in severe disease. The multidirectional nature of the relationship of the Imax values and lung function parameters was revealed. The negative correlation between these indicators was established in moderate COPD; a positive relationship was found in severe disease.

A positive relationship between the AOA value and all the studied lung function parameters in patients with severe COPD was established. The increase in Imax/S (AOA) values occurred against the background of an increase in the IC level in GOLD II.

### 3.7. Analysis of Correlation between Oxidative Stress Indicators

The results of the association analysis between the concentration of LPO products and the studied CL were the following:

- The negative correlations between the values of Imax/S (AOA) and levels of SB and DC (*r* = −0.45*p* = 0.001 and *r* = −0.43*p* = 0.001, respectively)
- The negative relationship between the Imax value and the SB level (*r* = −0.38*p* = 0.002)
- The positive association of the DC concentration and the value of light sum (*r* = 0.41*p* = 0.001)

### 3.8. Associations between sCD95 Levels and Lung Function Parameters

An analysis of associations showed a positive correlation between serum and endobronchial sCD95 levels and the studied lung function parameters in all examined patients (Table 3).

### 3.9. The Relationship between the Studied Markers of Oxidative Stress and the Level of sCD95 in COPD Patients

The relationship between the sCD95 level and the studied OS indicators was revealed only in patients with severe airway obstructive disorders (Table 4). In this group of patients, a decrease in the concentration of sCD95 both in the bloodstream and in the EBC occurs against the background of an increase in the SB level, as well as a decrease in DC concentration and Imax and Imax/S (AOA) values.

## 4. Discussion

In our work, we established a change in the concentration of the studied molecular products of lipid peroxidation (SB, DC) and CL indicators (Imax, S, Imax/S) in the blood serum of patients with COPD depending on the severity and period of the disease.

Increased DC and SB levels were found in all examined COPD patients compared with healthy nonsmoking volunteers. The established negative correlations between DC concentration and FEV1 and FEV1/FVC values in all studied patients may indicate the relationship between the activity of the initial processes of lipoperoxidation and the degree of airway obstruction violation. It is generally accepted that diene conjugates (DCs) are formed at the initiation stage of LPO reaction chain. At the final stage of lipid peroxidation, the resulting malondialdehyde (MDA) interacts with free amino groups to form the fat-soluble fluorescent and end product LPO—Schiff's base (SB) [35]. Quantifying the levels of initial and end products (DC and SB) allows assessing the activity and direction of LPO processes. The obtained high rates of diene conjugates and Schiff bases indicate an intensification of LPO processes in moderate and severe COPD. Our results are consistent with research data, which found an increase in the serum concentration of lipid peroxidation products such as MDA in COPD patients [6–8, 21].

The present work revealed that the maximum concentration of DCs was typical for patients with moderate COPD during the exacerbation period. Based on the results obtained, it can be assumed that activation of the initial lipid peroxidation stage predominates at this disease stage. The high concentration of DCs decreased with the increasing severity of COPD. The decline in these molecules level may be associated with the subsequent transformation of diene conjugates into secondary and end LPO products, depletion of oxidized substrates, and/or a decrease in the intensity of free radical processes against the background of chronic inflammation progression and under conditions of increasing hypoxia. This assumption is confirmed by results demonstrating an increase in SB concentration and a decrease in the DC/SB ratio in COPD patients with severe obstructive airway disorders. These results may indicate the predominance of the final stage of lipid peroxidation processes and the accumulation of end LPO products in GOLDIII. Under physiological conditions, SB transfers toxic and unstable products of lipid peroxidation metabolism (aldehydes, etc.) to the path of further utilization. However, in high concentrations, SB can modify serum lipoproteins and have a destructive effect on cells due to the destruction of intermolecular cross-links of biopolymers and damage to membranes [36]. In this case, the accumulation of these end products of LPO can be one of the reasons for the dysregulation of reparative processes, the formation of lung tissue remodeling, and the intensification of chronic inflammatory processes [37].

The revealed negative relationship between Schiff base level and FEV1/FVC values in GOLDIII indicates the mutual influence of the intensity of systemic oxidative stress and the functional capacity of the lungs under conditions of increasing chronic inflammation severity. The results obtained make it possible to consider the excessive accumulation of Schiff bases as an additional diagnostic criterion for an unfavorable course of COPD.

The CL parameters (Imax, S) also exceeded the control group's values and depended on disease severity. The intensity of induced CL (Imax) in the blood serum reflects the potential ability of lipid substrates to form highly active electron-excited products [22, 23]. Accordingly, the registered increased Imax values confirm the activation of free radical reactions in all examined COPD patients. At the same time, the inhibition of CL intensity was revealed in COLDIII compared to patients with moderate COPD. The established decrease in the CL response against the background of increased violations of airway obstruction is confirmed by a positive correlation between Imax and FEV1 and FEV1/FVC in GOPDII. The recorded decline in Imax values with a growth in the severity of the disease may be associated with an increasing deficiency of lipid substrates for oxidation due to prolonged activation of free radical reactions. This assumption is confirmed by the increased formation and accumulation of end products (SB) of LPO, which are not capable of oxidation, as well as a decrease in the concentration of DCs in severe COPD compared with GOLDII patients.

The revealed negative correlation between the SB and Imax levels also indicates a decrease in the CL intensity against the background of an increase in the concentration of lipid peroxidation end products. Thus, lower Imax values in GOLDIII relative to patients with moderate COPD may indicate an unfavorable course of inflammation in patients with this disease.

The value of the light sum (S) increased with the progression of COPD severity. It should be noted that in the examined patients GOLDII and GOLDIII, the S level and DC concentration were significantly higher during the exacerbation than in the stable period. In this case, the results obtained allow us to consider the DC and light sum values as prognostic markers of an intensification in the inflammatory process in COPD.

The light sum value reflects the concentration of free radicals and is inversely proportional to the activity of the antioxidant system. Accordingly, the assessment of antioxidant activity (AOA) in the blood serum was determined by the Imax/S ratio. The observed increase in Imax/S (AOA) level against the background of high values of the intensity of CL in the GOLDII group is probably a response of the protective mechanism of antioxidant protection to the activation of FRO processes in patients with moderate obstructive airway disorders. At the same time, a decrease in Imax/S (AOA) values in GOLD III may indicate a depletion of the components of the general antioxidant defense under the condition of systemic chronic inflammation progression. The revealed positive correlations between Imax/S and FEV1 and FEV1/FVC in severe COPD confirm the association between a decrease in antioxidant potential and the formation of persistent ventilation restrictions in the growth of disease severity.

Thus, the detected increase in SB concentration, as well as a decrease in the level of DC, Imax, and Imax/S (AOA) in GOLDIII patients, may indicate a violation of the feedback principle in the proantioxidant system and dysregulation of FRO processes in severe inflammation.

It is known that OS has a wide range of biochemical and pathophysiological effects that affect the regulatory processes of inflammation in COPD, including apoptosis [38]. In this regard, we considered it expedient to study the features of changes in sCD95 concentrations depending on the stages and period of COPD as well as and the relationship of the studied FRO parameters with this apoptosis marker level. Previously, it was shown that an increase in the severity in patients with exacerbation of COPD is accompanied by a decrease in the level of sCD95 in the blood serum and EBC [3]. In the present work, we evaluated the change in this apoptosis marker level compared to the exacerbation and the stable phase at different disease stages. As a result, we did not find differences in sCD95 concentration in studied COPD patients depending on the disease period. In addition, the decrease in this apoptosis marker concentration was established in the tested biological fluids with the progression of inflammation, which was confirmed by a positive correlation between spirometric parameters and concentrations of these molecules in both moderate and severe COPD. Functionally, sCD95 competes with the CD95 membrane-localized receptor for Fas ligand binding and thus can inhibit CD95-mediated apoptosis [39]. Thus, the soluble form of this apoptotic marker can participate in the preservation of cellular homeostasis during the normally developing process of programmed cell death. In this case, the decrease in sCD95 level in patients with severe COPD creates conditions for more effective implementation of Fas-dependent apoptosis. This change in sCD95 concentration can increase the apoptosis progression of pathogenetically significant cells and promote the development of destructive processes in the lung tissue and the whole body in GOLDIII patients.

Association analysis showed multiple correlations between serum and endobronchial sCD95 levels and the studied OS markers values in patients with severe COPD. The revealed relationships may indicate indirect participation of the tested OS indicators in activating programmed cell death processes through Fas-mediated mechanisms. These mechanisms may underlie the development of reparative disorders and, as a result, the lung fibrosis formation and pathophysiological manifestations, severe inflammation in GOLDIII patients.

In our work, we also established the positive correlations between the levels of sCD95, Imax/S (AOA), and such a spirometric indicator as to the inspiratory capacity (IC) in all examined COPD patients. Also, the decline in IC values was accompanied by an increase in SB concentration in severe disease. The decrease in IC level is associated with pulmonary hyperinflation development, increased hypoxia, and loss of elasticity of the lung tissue in COPD.

Thus, the obtained data may indicate that a decrease in the antioxidant potential, a violation of the regulation of LPO processes, and an intensification in apoptotic reactions contribute to structural and functional changes in the respiratory tract, as well as the formation of hypoxemia and impaired gas exchange in severe inflammation in COPD patients.

A limitation of the presented results in this work may be the approaches to the interpretation of the obtained data in EBC. To date, there are various methodological approaches for the determination of analytes in exhaled breath condensate. EBC is a liquid formed as a result of cooling and subsequent condensation of exhaled breath. From a physical point of view, the exhaled breath is an aerosol or an aerodisperse system consisting of a gaseous dispersion medium and a liquid dispersed phase suspended in it (aerosol particles). In other words, EBC is a suspension of liquid particles in a gas [40]. One of the main components of exhaled breath is water vapor. The concentration of water vapor in exhaled breath is a fairly constant value, little dependent on environmental parameters [41]. The levels of nonvolatile components of exhaled air can vary depending on a number of factors, such as the exhalation rate, the state (diameter) of the airways, and pathophysiological processes in airway lining fluid (ALF) [40–46]. In several works, attempts have been made to standardize the assessment of nonvolatile compounds in EBC, by taking into account the degree of dilution of EBC. So, the dilution of the EBC was estimated based on the measurement of its conductivity [47, 48]. In the works [49, 50], the level of urea in EBC was used as a marker of dilution. In another study [51], the assessment of the degree of dilution was studied by the concentrations of K+, Na+, Mg2+, Ca2+, the total level of cations, urea level, and the EBC conductivity. However, until now, a unified approach to standardizing the assessment of nonvolatile compounds in EBC has not been found. In our opinion, the concentrations of the analytes considered in these studies may vary depending on the pathophysiological processes in the airway lining fluid, especially in lung diseases. Accordingly, these approaches may incorrectly display the dilution rate of the EBC and require further study. Earlier, in our work, a methodology was proposed for standardizing the procedure for collecting EBC and evaluating nonvolatile components in ALF based on taking into account the concentration of aerosol particles in exhaled breath [52]. Therefore, in this work, we proceeded from the assumption that the concentrations of nonvolatile components in the EBC are indicators reflecting the pathophysiological processes in the airway lining fluid of patients.

## 5. Conclusion

The findings indicate not only the participation of OS in the development of COPD but also show the importance of determining the degree of FRO activity and antioxidant potential, taking into account the severity of impaired ventilation function of the lungs. A complex evaluation of OS indicators in COPD patients, including the determination of the concentration of DC and SB, the values of CL parameters in the blood serum, allowed us to clarify the activity of systemic FRO reactions depending on the severity of airway obstruction degree and disease periods.

Combining an increase in SB concentration, a decrease in DC, Imax, Imax/S (AOA), and sCD95 levels in GOLDIII patients allows us to consider these indicators as additional quantitative indicators for predicting the disease severity.

The exacerbation of the disease was characterized by the most significant increase in DC level in the blood serum compared to the stable period of the disease, which makes it possible to use the concentration of this LPO product as potential monitoring markers of inflammation intensification in COPD.

The established numerous associations between the levels of the studied LPO products, the CL, sCD95, and spirometric parameter values in COPD patients indicate the mutual influence of the intensity of systemic oxidative stress, the processes of apoptosis, and the functional capacity of the lungs. Also, the obtained correlations make it possible to consider the studied indicators of OS and apoptosis as laboratory-diagnostic and prognostic markers of systemic and local chronic inflammation progression in COPD.

The revealed relationship between the values of sCD95 and OS markers in GOLD III patients may indicate the possible participation of LPO products and FRO processes in the forming soluble CD95 and apoptosis processes activity both in the respiratory tract and whole body in severe obstructive airway violation.

The results obtained make it possible to clarify and supplement the influence of systemic oxidative stress and apoptosis processes on the development of pathophysiological features in moderate and severe forms of COPD and, accordingly, to improve the efficiency of diagnosis and treatment of the disease.

## Figures and Tables

**Table 1:** Characteristics of COPD patients and healthy nonsmokers included in the study.

	Healthy nonsmokers	COPD			
		Moderate		Severe	
	1	2	3	4	5
Subjects (*n*)	21	Exacerbation (*n* = 25)	Stable (*n* = 27)	Exacerbation (*n* = 29)	Stable (*n* = 24)
Sex, male/female	15 (71%)/6 (29%)	18 (72%)/7 (28%)	22 (81%)/5 (19%)	26 (90%)/3 (10%)	20 (83%)/4 (17%)
Age (years)	50.4 ± 9.7	52.6 ± 7.9	49.5 ± 5.6	58.3 ± 4.2	55.2 ± 6.5
Smoking pack-years	0	36.5 ± 4.8	34.3 ± 5.3	42.6 ± 3.9	39.6 ± 5.1
FEV_1_ % pred	101.3 ± 5.3	59.1 ± 7.1 *p*_1_ = 0.001	65.4 ± 8.2 *p*_1_ = 0.001 *p*_2_ = 0.01	36.5 ± 5.2 *p*_1_ = 0.001 *p*_2_ = 0.001 *p*_3_ = 0.001	42.5 ± 9.8 *p*_1_ = 0.001 *p*_2_ = 0.001 *p*_3_ = 0.001 *p*_4_ = 0.02
FEV_1_/FVC %	104.2 ± 3.7	55.7 ± 9.2 *p*_1_ = 0.001	63.8 ± 11.3 *p*_1_ = 0.001 *p*_2_ = 0.01	46.5 ± 8.2 *p*_1_ = 0.001 *p*_2_ = 0.001 *p*_3_ = 0.001	51.9 ± 10.5 *p*_1_ = 0.001 *p*_2_ = 0.001 *p*_3_ = 0.001 *p*_4_ = 0.04
Inspiratory capacity IC (%)	108.3 ± 3.5	64.5 ± 6.1 *p*_1_ = 0.001	70.8 ± 9.1 *p*_1_ = 0.001 *p*_2_ = 0.01	59.6 ± 12.6 *p*_1_ = 0.001 *p*_2_ = 0.001 *p*_3_ = 0.001	68.5 ± 14.1 *p*_1_ = 0.001 *p*_2_ = 0.001 *p*_3_ = 0.001 *p*_4_ = 0.01
COPD medication	—				
LAMA		2 (8%)	4 (14.8%)	—	—
LAMA+LABA		7 (28%)	13 (48.1%)	8 (27.6%)	6 (25%)
ICS+LABA+LAMA		16 (64%)	10 (37%)	21 (77.8%)	18 (75%)
SCS		9 (36%)	—	29 (100%)	—

Data were presented as mean ± SD. COPD: chronic obstructive pulmonary disease; pack-years: number of cigarette packs per day multiplied by the number of smoking years; FEV_1_: forced expiratory volume in one second; % pred: % predicted; FVC: forced vital capacity; IC: inspiratory capacity (%); LAMA: long-acting muscarinic antagonists; LABA: long-acting *β* agonists; ICS: inhaled corticosteroids; SCS: systemic corticosteroids.

**Table 2:** Changes in indicators of oxidative stress in patients with moderate to severe COPD in different periods of the disease.

	Healthy nonsmoking volunteers	GOLDIIex	GOLDIIst	GOLDIIIex	GOLDIIIst
	1	2	3	4	5
Schiff bases (RU)	3.2 ± 0.25	4.3 ± 1.2 *p*_2−1_ = 0.01	3.8 ± 0.8 *p*_3−1_ = 0.03 *p*_3−2_ > 0.05	5.7 ± 2.3 *p*_4−1_ = 0.01 *p*_4−2_ = 0.04 *p*_4−3_ = 0.001	4.7 ± 1.7 *p*_5−1_ = 0.01 *p*_5−2_ > 0.05 *p*_5−3_ = 0.001 *p*_5−4_ > 0.05
Diene conjugates (RU)	0.165 ± 0.01	0.26 ± 0.046 *p*_2−1_ = 0.001	0.202 ± 0.028 *p*_3−1_ = 0.001 *p*_3−2_ = 0.048	0.209 ± 0.02 *p*_4−1_ = 0.001 *p*_4−2_ = 0.042 *p*_4−3_ = 0.385	0.19 ± 0.03 *p*_5−1_ = 0.001 *p*_5−2_ = 0.001 *p*_5−3_ > 0.05 *p*_5−4_ = 0.026
Diene conjugates/Schiff base	0.0515 ± 0.02	0.06 ± 0.015 *p*_2−1_ = 0.032	0.0664 ± 0.035 *p*_3−1_ = 0.035 *p*_3−2_ > 0.05	0.0368 ± 0.008 *p*_4−1_ = 0.001 *p*_4−2_ = 0.001 *p*_4−3_ = 0.001	0.04 ± 0.017 *p*_5−1_ = 0.001 *p*_5−2_ = 0.003 *p*_5−3_ = 0.001 *p*_5−4_ > 0.05
Imax (RLU)	0.061 ± 0.029	0.265 ± 0.19 *p*_2−1_ = 0.001	0.23 ± 0.13 *p*_3−1_ = 0.01 *p*_3−2_ > 0.05	0.18 ± 0.1 *p*_4−1_ = 0.001 *p*_4−2_ = 0.01 *p*_4−3_ > 0.05	0.13 ± 0.07 *p*_5−1_ = 0.01 *p*_5−2_ = 0.01 *p*_5−3_ = 0.031 *p*_5−4_ > 0.05
S	0.75 ± 0.18	2.13 ± 0.48 *p*_2−1_ = 0.016	1.75 ± 0.23 *p*_3−1_ = 0.002 *p*_3−2_ = 0.01	3.1 ± 0.46 *p*_4−1_ = 0.001 *p*_4−2_ = 0.001 *p*_4−3_ = 0.001	2.09 ± 0.34 *p*_5−1_ = 0.047 *p*_5−2_ > 0.05 *p*_5−3_ = 0.001 *p*_5−4_ = 0.001
Imax/S	0.08 ± 0.01	0.12 ± 0.05 *p*_2−1_ = 0.041	0.13 ± 0.05 *p*_3−1_ = 0.046 *p*_3−2_ > 0.05	0.058 ± 0.01 *p*_4−1_ = 0.034 *p*_4−2_ = 0.01 *p*_4−3_ = 0.01	0.062 ± 0.01 *p*_5−1_ = 0.049 *p*_5−2_ = 0.01 *p*_5−3_ = 0.01 *p*_5−4_ > 0.05

Data were presented as mean ± SD.

**Table 3:** Correlations between oxidative stress markers and lung function parameters in patients with moderate to severe COPD.

	GOLDII			GOLDIII		
	FEV_1_ (%)	FEV_1_/FVC (%)	IC	FEV_1_ (%)	FEV_1_/FVC (%)	IC
Diene conjugates	*r* = −0.47 *p* = 0.026	*r* = −0.43 *p* = 0.045	*r* = −0.33 *p* = 0.18	*r* = −0.45 *p* = 0.01	*r* = −0.43 *p* = 0.03	*r* = −0.41 *p* = 0.09
Schiff base	*r* = 0.08 *p* = 0.68	*r* = 0.32 *p* = 0.18	*r* = −0.39 *p* = 0.11	*r* = −0.3 *p* = 0.18	*r* = −0.42 *p* = 0.03	*r* = −0.5 *p* = 0.04
Imax	*r* = −0.54 *p* = 0.02	*r* = −0.6 *p* = 0.01	*r* = 0.103 *p* = 0.69	*r* = 0.36 *p* = 0.04	*r* = 0.5 *p* = 0.03	*r* = 0.13 *p* = 0.57
Imax/S	*r* = 0.32 *p* = 0.11	*r* = 0.27 *p* = 0.19	*r* = 0.63 *p* = 0.01	*r* = 0.47 *p* = 0.04	*r* = 0.72 *p* = 0.01	*r* = 0.52 *p* = 0.02
sCD95 (serum)	*r* = 0.61 *p* = 0.01	*r* = 0.5 *p* = 0.02	*r* = 0.62 *p* = 0.01	*r* = 0.43 *p* = 0.02	*r* = 0.45 *p* = 0.02	*r* = 0.6 *p* = 0.002
sCD95 (EBC)	*r* = 0.28 *p* = 0.24	*r* = 0.64 *p* = 0.01	*r* = 0.58 *p* = 0.04	*r* = 0.65 *p* = 0.001	*r* = 0.46 *p* = 0.04	*r* = 0.53 *p* = 0.03

*r*: correlation coefficient; FEV_1_: forced expiratory volume in 1 second; % pred: % predicted; FVC: forced vital capacity; IC: inspiratory capacity (%); EBC: exhaled breath condensate.

**Table 4:** Correlations between the studied system markers of oxidative stress and sCD95 levels in serum and EBC in patients with moderate to severe COPD.

	sCD95 (serum) GOLDII	sCD95 (EBC) GOLDII	sCD95 (serum) GOLDIII	sCD95 (EBC) GOLDDIII
Schiff base	*r* = 0.27 *p* = 0.22	*r* = −0.27 *p* = 0.24	*r* = −0.39 *p* = 0.038	*r* = −0.46 *p* = 0.02
Diene conjugates	*r* = −0.3 *p* = 0.25	*r* = 0.25 *p* = 0.27	*r* = 0.37 *p* = 0.046	*r* = 0.23 *p* = 0.35
Imax	*r* = −0.13 *p* = 0.54	*r* = −0.06 *p* = 0.79	*r* = 0.37 *p* = 0.046	*r* = 0.58 *p* = 0.001
Imax/S	*r* = 0.3 *p* = 0.25	*r* = 0.3 *p* = 0.25	*r* = 0.38 *p* = 0.045	*r* = 0.42 *p* = 0.02

*r*: correlation coefficient; EBC: exhaled breath condensate.

## Data Availability

The data used to support the findings of this study are available from the corresponding author upon request.
